# Validation of the Arabic Version of the Epworth Sleepiness Scale among the Yemeni Medical Students

**DOI:** 10.1155/2020/6760505

**Published:** 2020-02-29

**Authors:** Bothaina Ahemd Attal, Fawziah Kassim Al-Ammar, Mohammed Bezdan

**Affiliations:** ^1^Community Medicine Department, Faculty of Medicine and Health Sciences, Sana'a University, Sana'a, Yemen; ^2^Educational and Psychological Sciences Department, Faculty of Education and Languages, Amran University, Amran, Yemen; ^3^Al Thawra Teaching Hospital, Sana'a, Yemen

## Abstract

The study was conducted with the aim to assess the psychometric measures of an adapted Arabic version of the Epworth Sleepiness Scale (ESS) among medical students at Sana'a University, Yemen. The cross-sectional study targeted 360 students (males: 176; females: 184) from the preclinical 3^rd^ year (*N*: 197) and the final clinical year (*N*: 163). Participants self-filled an Arabic and slightly modified version of the 8-item Epworth Sleepiness Scale. Exploratory Principal Component Analysis (PCA) and Confirmatory Factor Analyses (CFA) were conducted on two equal subsets of the sample (*N*: 180 each). The PCA yielded a two-dimension model subsequently confirmed by factor analysis. The first dimension was grouped on three items while the second dimension had five items reflecting the respondents' propensity to sleep during “interactive situations” and “sitting and lying,” respectively. The model had an acceptable goodness of fit measures for the overall ESS (CMINDF = 2.362, CFI = 0.91, IFI = 0.92) and acceptable reliability indicators (factor 1 *α* = 0.65, factor 2 *α* = 0.62). However, due to weak variance explanation (0.07) of item 6 (sitting and talking) in factor 1, analysis was repeated excluding this item. The 7-item model was also two-dimensional, valid, and reliable. The reliability indicators were acceptable with *α* = 0.65 for factor 1 (4 items of interactive situations) and 0.62 for factor 2 (3 items of sitting) and overall *α* = 0.68. Overall, the ESS is a useful tool. Factor analysis produced a two-factor model of 7 items with good validity and reasonable reliability that can be used in diagnosing daytime sleepiness among young Yemeni adults.

## 1. Introduction

Excessive Daytime Sleepiness (EDS) is described by Johns [1] as “a symptom arising at any time from an excessive propensity to become drowsy or to fall asleep, when the intention and expectation is to remain awake and alert at the time.” Several methods and tools were developed to assess sleepiness whether objectively using standardized methods or subjectively by seeking the individual's account [1] of their sleepiness. The Epworth Sleepiness Scale (ESS), a subjective assessment tool, was first developed in 1990 [2] and modified in 1997 to assess EDS among adults [3]. Since then, the scale has been used in general [4] and clinical populations [5, 6] and was effectively adapted to children and adolescents [7, 8].

The tool is a simple, short, and self-administered questionnaire. Respondents rate on a 4-point Likert scale (0-3) their usual chances of having dozed off or fallen asleep while engaged in eight different situations such as sitting and reading, lying down after lunch without alcohol, and being a passenger in a car for an hour without a break. These situations pertain to body positions and activities that have varying effects on an individual's tendency to doze off or, in other words, varying degrees of somnificity [9, 10]. The sum of the integer rating of these items yields the total ESS score. This score ranges between 0 and 24 and gives an estimate of the “average sleep propensity” (ASP) of a person across the included interactive situations. The “normal” ASP or ESS score ranges from zero to 10, a value estimated and confirmed by data from the general population in several countries [4, 11, 12]. Higher ESS scores represent increasing levels of ASP or EDS.

The scale was originally developed in English but was eventually translated into a number of languages and used in different cultures [6, 12–16]. The scale correlates significantly with the objective tests such as the Multiple Latency Sleep Test, which is considered a gold standard test [2, 17]. It has shown acceptable to robust psychometric properties [18] and high sensitivity and specificity in its original [7, 19] and translated versions [6–8, 12, 13, 15, 20].

Evaluating sleepiness and sleep disorders subjectively entails that the tool must correspond to the participants' culture and language. In this study, the ESS was adapted, translated into Arabic, and administered to assess EDS among medical students in Sana'a University, Yemen. In comparison to the other subpopulations, medical students were reported to be more prone to sleep disorders which can undermine their well-being [21] and academic performance [22, 23]. This paper presents an analysis of the psychometric measures of the Epworth Sleepiness Scale in the Adapted Arabic-Yemeni version.

## 2. Materials and Methods

### 2.1. Study Design

A cross-sectional study was conducted in October 2017 at the Faculty of Medicine and Health Sciences (FMHS), Sana'a University. The faculty, established in 1989, offers a 6-year-long Bachelor of Medicine and Bachelor of Surgery (MBBS) degree program divided into 3 preclinical years followed by another 3 clinical levels. Yemen, on the other hand, is an Arab country of around 28 million predominantly Muslim population. The country is under war since March 2015 which caused disruption of all aspects of life including the university's administrative and the teaching process.

### 2.2. Study Population and Sample

The study targeted 370 out of 705 medical students at the FMHS. Students were selected by simple random sampling according to the sex and educational level (the preclinical 3rd year and the final 6th year). Out of 370, 10 cases were omitted because of missing item responses. Missing one or more of the 8 item scores render the ESS total score invalid as it is not feasible to interpolate missing item scores [24]. Respondent's characteristics are presented in Table 1.

### 2.3. Study Questionnaire

Participants responded to two parts of a questionnaire: the first part covers the demographic and life style information such as subjective financial status, subjective health status, and chewing Khat (plant with amphetamine-like effects commonly used as a mild stimulant in some African countries and Yemen) [25] and having a job besides studying. The second part of the questionnaire comprises the adapted and translated version of the 8-item ESS.

In the current study, the original scale was forward-backward translated into Arabic following the translation steps adapted by Brislin et al. [26]. The questions were retained as is except question 7 where “without alcohol” was removed from the statement to be “sitting quietly after lunch.” Alcohol and questions on alcohol are culturally not acceptable in Yemen similar to other Muslim countries. The questionnaire was finally pretested in a sample of 10 students and modified accordingly. Annex 1 shows the original and Arabic versions of the ESS.

### 2.4. Data Analysis

Multistage analysis was used to assess the scale's validation measures and to explore its factorial structure. Therefore, the data pertaining to 360 completed responses were divided randomly and equally. An exploratory Principal Component Analysis (PCA) was run on the first half of the sample (*N*: 180) followed by Confirmatory Factor Analysis (CFA) on the other data subset.

The PCA was run to identify the underlying dimensions of the ESS among the Arabic speakers, testing the intercorrelation among the ESS items. Initially, the two measures of Kaiser-Meyer-Olkin (KMO) and Bartlett's Test of Sphericity were examined to determine the sampling adequacy for structure detection. Then, the PCA analysis with Varimax rotation and eigenvalues greater than one was applied to determine the factor structure of the ESS. On the other hand, the CFA was used to validate the hypothesized PCA-generated ESS model. PCA indicated a two-factor structure of the ESS (Table 2). Using AMOS, maximum likelihood estimation with estimation means and intercepts were run to generate the estimates of the full-fledged measurement model [27, 28]. To assess the fit of the 8-item measurement model using CFA, a number of descriptive statistics were considered: the minimum value of the discrepancy between the observed data and hypothesized model and the chi-square (CMIN) index divided by the degree of freedom (CMIN/DF) considering values between 2 and 5 as acceptable [29, 30]. The Comparative Fit Index (CFI) and Incremental Fit Index (IFI) were assessed and both should exceed the threshold of 0.90. Finally, the value of 0.08 and less was acceptable as a measure of the Root Mean Square Error of Approximation (RMSEA) [30–32].

Finally, Cronbach alpha [33] was used to measure the scale reliability so as to produce a complete the picture of the ESS psychometric characteristics. SPSS v.24 [34] was used for data cleaning, simple descriptive statistics, and the PCA while CFA was run using AMOS v.23 [27].

### 2.5. Ethical Consideration

Ethical clearance for the study was obtained from the Faculty of Medicine and Health Sciences, Sana'a University. Before data collection, students received information about the purpose and content of the study and consent for participation was verbal. No personal identifiers were included in the questionnaire. Anonymity and confidentiality were maintained on collecting the answered questionnaires.

## 3. Results

### 3.1. Study Participants

Table 1 shows the respondents' characteristics and the Epworth Sleepiness Scale results (ESS). The sample was divided equally between males (*N*: 176, 49%) and females (184, 51%) with mean age of 23.4 years (SD = 0.50). The majority of participants were single (*N*: 293, 81.4%) the remaining were either married (14%) or others (5%). The majority rated their financial status as good (*N*: 135, 37.5%) or managing (*N*: 203, 56.5%), while 6% were poor. Almost half of the students (45%) qualified to the diagnosis of excessive daily sleepiness with a mean ESS score of 9.7 (±SD: 4.2), just below the excessive sleepiness point of 10.

### 3.2. Principle Component Analysis (PCA)

PCA was conducted on the first set of 180 participants to identify the factorial structure of the ESS among Yemeni students of the FMHS, Sana'a University. Testing certain statistical assumptions showed that the intervariable correlation among the 8 items supported the use of PCA; the size of correlation does not exceed the criteria score (*r* ≤ 0.30), and the two measures of Kaiser-Meyer-Olkin (KMO) indicate that the overall Measure of Sampling Adequacy (MSA) was statistically adequate (0.65). More precisely, Bartlett's Test of Sphericity indicated a statistically significant correlation among the variables (*χ*^2^ (28) = 256.945, *p* = 0.001). Using Varimax rotation, two factors were extracted following the criteria of Hair et al. [30] regarding the sample size and items.  Table 2 shows the findings. The eight items with eigenvalues greater than one explained 49.4% of the total variance. The loading values ranged from 0.86 (watching TV) to 0.46. (sitting and talking to someone). Considering a sample size of 180 students, the first factor loading of the three items is significant. Guided by the criteria of Hair et al. [30], the content of the three items reflect *interactive situations*. On the other hand, the remaining five items were loaded on factor 2 with values ranging between 0.80 (in a car, while stopped for a few minutes in traffic) and 0.46 (sitting and talking to someone). These factors have *sitting and lying* as a common feature.

### 3.3. Confirmatory Factor Analysis

Confirmatory Factor Analysis (CFA) was applied on the second subset of respondents to test the hypothesized PCA model. CFA was run, with a maximum likelihood estimation with means and intercepts estimation, to generate the full-fledged measurement model of the ESS.

**Figure 1:**
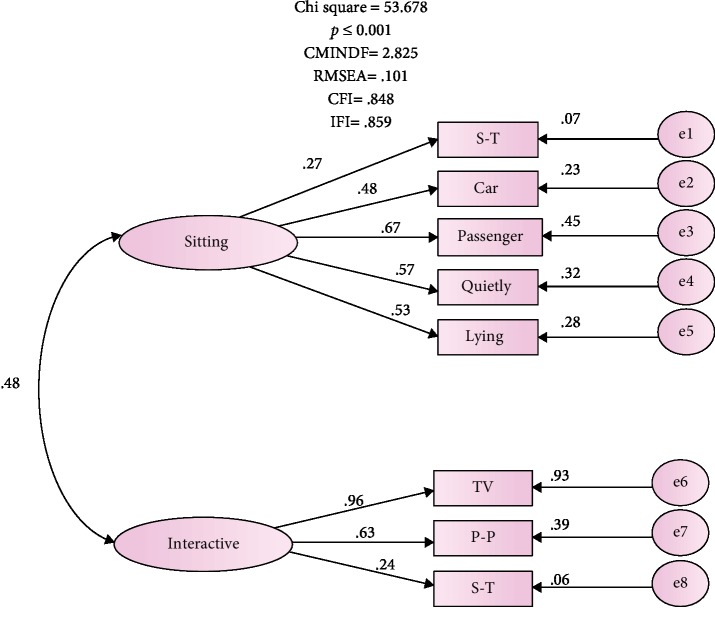
The hypothesised model of the 8-item ESS. e2-e8: car-S-R represent error variances; double-headed arrows represent correlation between two factors; single-headed arrows from factors depict factor loading to items. S-T: sitting and talking; car: in a car while stopped a few minutes; passenger: as a passenger in a car for an hour without a break; quietly: sitting quietly after a lunch; lying: lying down to rest in afternoon; TV: watching TV; P-P: public places; S-R: sitting and reading.

The results report of the CFA was free of offending estimate. The loading estimates ranging 0.24-0.96 and a *t* value loading greater than 2.0 were statistically significant, *p* ≤ 0.001 (Figure 1). The correlation between the two latent factors of “interactive situations” and “sitting situation” was good and substantiated (*r* = 0.48). However, the results of the overall fit of the ESS model was not equally encouraging. This is based on the observation that the significant value of chi-square indicates discrepancy between the hypothesized model and the proposed one (*χ*^2^ (19) = 53.678, *p* = 0.001). Other findings point towards significant discrepancies between the observed covariance and the implied matrices, reflecting a possible fit problem. The value of CMIN/DF was 2.83, higher than the recommended cutoff score of 2. In addition, the fit indicators of Comparative Fit Index (CFI) and Incremental Fit Index (IFI) did not reach the 0.90 important threshold for model fit [30]. Also, the Root Mean Square Error of Approximation (RMSEA) 0.101 exceeded the recommended range (0.05–0.08) of the accepted fit [29, 30, 32, 35].

The model's lack of fit can be explained by one possible reason. The square multiple correlation (SMC) for item 6 “sitting & talking,” although significantly loaded on factor 2, was only 0.070. The fact that the factor 2 “sitting” extracted only about 0.07% of the variance in item 6 undermines the reliability of the factor. Thus, the hypothesized model was revised excluding this item in the subsequent analysis.  Table 3 shows the ESS model in both the hypothesized and revised versions excluding the “sitting and talking” item in the latter.

**Figure 2:**
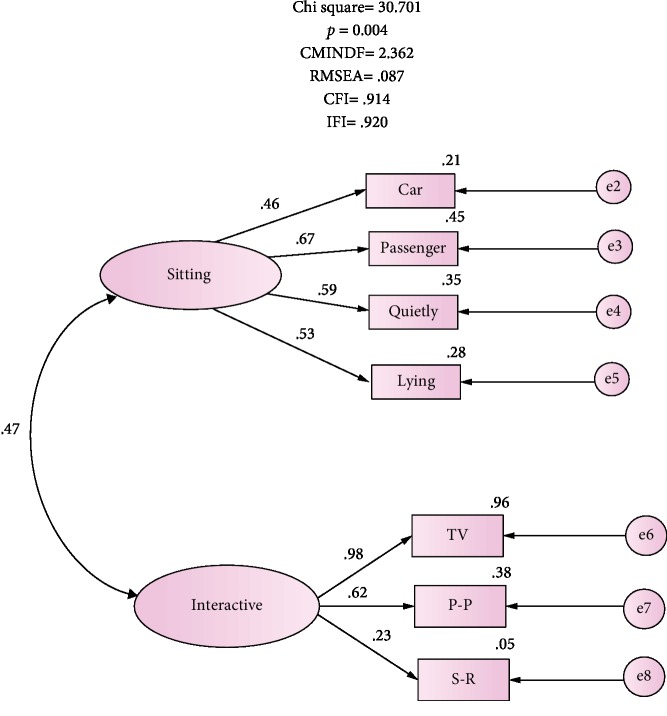
The revised factorial structure of the 7-item ESS. e2-e8: car-S-R represent error variances; single-headed arrows from factors depict factor loading to items; car: in a car while stopped a few minutes; passenger: as a passenger in a car for an hour without a break; quietly: sitting quietly after a lunch; lying; lying down to rest in afternoon; TV: watching TV; P-P: public places; S-R: sitting and reading.

To validate the revised ESS model, a second Confirmatory Factor Analysis was applied on the second sample (*n* = 180). The parameter estimates were free from negative values and error variances; the value of factor loading exceeded the value of 2.0. The overall fit of the 7-item measurement model is shown in Figure 2. The goodness of fit for the revised model is consistent with the data sample yielding fit indices of CMINDF = 2.4, CFI = 0.914, IFI = 0.920, and RMSEA = 0.087 based on previously recommended indices [29, 30]. In addition, omitting item 6 improved the fit indices: RMSEA increased from 0.014 to 0.087, CFI rose by 0.066 to reach 0.914, and IFI increased by 0.061 to 0.920. Internal consistency of the factors remained the same *α* values of 0.65 for factor 1 (4-item sitting situation) and 0.62 for factor 2 (3-item interactive situations). The revised 7-item ESS model had an acceptable *α* of 0.68 compared to 0.65 for the 8-item scale.

## 4. Discussion

This study examined the psychometric measures of an adapted Arabic version of the 8-item Epworth Sleepiness Scale (ESS) among 360 male and female Yemeni medical students from the preclinical and clinical levels at the Faculty of Medicine and Health Sciences, Sana'a University. Multistage analysis of exploratory Principle Component Analysis followed by Confirmatory Factor Analyses were applied together with assessment of the reliability. The ESS loaded on two factors in the Yemeni context retaining 7 items with a Cronbach's alpha of 0.678 for the whole scale, and a corrected item-total correlations between 0.194 and 0.51 (Table 2). The ESS was validated in different cultures and contexts and was found to have good reliability with Cronbach's alpha ranging between 0.69 and 0.88 [18]. With 0.678 Cronbach's alpha, our study is consistent with the finding that the scale's internal consistency tends to be lower among the nonclinical respondents such as students [35] and elderly women [36] compared to individuals with sleep disorders [12, 13, 35].

On the other hand, the ESS has been described as a unidimensional 8-scale by its developer [35] and other authors from different settings [8, 17, 18]. Few studies reported extracting two factors though, such as a study that assessed an Arabic ESS version among students in Sudan [15] and an Iranian version that was tested among patients with sleep disorders [37]. According to the content of the items loaded on each factor, authors suggest that factor 1 with 4 items represents an individual's propensity to sleep in “interactive situations” while factor 2 of 3 items reflects this propensity in “sitting and lying.” Item 6 of “sitting and talking” had poor, although significant, loading on factor 2, and a low corrected item-total correlation (0.239) caused by low square multiple correlation (SMC), explaining 0.07% of the factor variance. We repeated the Confirmatory Factor Analysis excluding this item and that produced a reliable 7-item scale (*α* = 0.68) with better fit indices at CMINDF = 2.4, CFI = 0.914, IFI = 0.920, and RMSEA = 0.087 (*χ*^2^ = 30.7, *p* = 4) (Figure 2). The “sitting and talking” item is a situation of low somnificity [10, 24]; i.e., there is a low probability of falling asleep while sitting and talking to someone, the fact which causes poor loading on the ESS especially among the nonclinical samples [18] such as ours.

This improvement of the scale measures proposes that sleepiness can be measured among the Yemeni medical students with acceptable reliability and validity using 7 items rather than 8.

The findings have to be interpreted in light of few limitations. First, medical students tend generally to have lower quality of sleep and they may therefore have higher tendency towards daily sleepiness. In addition, daily sleepiness may be aggravated by the fact that the data were collected around the time of final exams where students may further reduce their duration of sleep. For practical reasons, the study has not included test-retest measures and thus we cannot report on this aspect of the psychometric measures. The modified Yemeni-Arabic version is in need for further examination among the general population as well as those with sleep disturbances. Having said these limitations, the study presents a robust analysis of the ESS in our context, and to our best knowledge, the current study is one of very few empirical investigations of the ESS psychometric characteristics in an Arabic speaking context, i.e., Yemeni adults' population.

## 5. Conclusion

The Yemeni version of the ESS loaded on two factors with 7 items with good psychometric characteristics, and such findings seem to be different from previous studies from other settings and participants. Overall, the ESS measure is a valid and reliable scale that can be used in diagnosing daytime sleepiness among young Yemeni adults.

## Figures and Tables

**Table 1:** Respondents' characteristics and Epworth Sleepiness Scale score (*N*: 360).

Characteristic (*N*: 360)	Frequency	Percentage
Age (mean years (±SD))	23.1	±0.5
Sex		
Male	176	49%
Female	184	51%
Level of education		
Third	197	54.7%
Sixth	163	45.3%
Marital status		
Single	293	81.4%
Married	50	13.9%
Other	8	2.2%
Place of residence (*N*: 357)		
With the family	255	71.4%
In campus	50	14%
Rented place	48	13.4%
Other	4	1.1%
Subjective economic status		
Good	135	37.5%
Managing	203	56.5%
Poor	22	6%
Having work beside the studies		
No	230	64%
Irregular	98	27.2%
Regular	32	8.8%
Chewing khat		
No	199	55.3%
Irregular	70	19.4%
Regular	91	25.3%
Smoking		
No	305	84.7%
Irregular	49	13.6%
Regular	6	1.7%
Epworth sleepiness score (mean score (±SD))	9.7	4.2
Excessive sleepiness (ESS score > 10)	161	45%

**Table 2:** Principal component analysis of ESS.

No.	Item	Correlated item-total correlation	Component 1	Component 2
1	Sitting and reading	0.217	0.456	
2	Watching TV	0.561	0.861	
3	Sitting, inactive in a public place (e.g., a theatre or a meeting)	0.534	0.819	
4	As passenger in a car for an hour without a break	0.421		0.591
5	Lying down to rest in the afternoon when circumstances permit	0.400		0.566
6	Sitting and talking to someone	0.239		0.462
7	Sitting quietly after lunch	0.502		0.698
8	In a car, while stopped for a few minutes in traffic variance explained	0.421		0.803
Cronbach's alpha (*α*)		0.65	0.65	0.62

**Table 3:** Standardized model and SMC^∗^ of the hypothesized and revised models.

Item	Label	Model			
		Hypothesized		Revised	
		Loading	SMC	Loading	SMC
Watching TV	TV	0.96	0.927	0.98	0.958
As a passenger in a car for an hour without a break	Passenger	0.67	0.454	0.67	0.447
Sitting, inactive in a public place (e.g., a theatre or a meeting)	Public places	0.63	0.392	0.62	0.379
Sitting quietly after a lunch	Quietly	0.57	0.282	0.59	0.350
In a car, while stopped for a few minutes in the traffic	Car	0.48	0.232	0.481	0.232
Lying down to rest in the afternoon when circumstances permit	Lying	0.53	0.282	0.53	0.278
Sitting and talking to someone	Sitting & talking	0.27	0.070	—	—
Sitting and reading	Sitting & reading	0.24	0.056	0.23	0.054
Cronbach's alpha (*α*)		0.65		0.68	

^∗^Square multiple correlation.

## Data Availability

Dataset generated and analyzed for the current study are available from the corresponding author on reasonable request.

## Supplementary Materials

Supplementary Materials Annex 1 containing the original and adapted Arabic versions of the Epworth Sleepiness Scale.
