# Supplementary material for: Validation of the Arabic Version of the Epworth Sleepiness Scale among the Yemeni Medical Students
Source: Sleep Disord. 2020 Feb 29;2020:6760505. doi: 10.1155/2020/6760505 (PMC7071801; doi:10.1155/2020/6760505)
Supplement: Supplementary Materials — Annex 1 containing the original and adapted Arabic versions of the Epworth Sleepiness Scale. [file 6760505.f1.docx]

**Annex 1**

**The original Epworth Sleepiness Scale**

### Epworth sleepiness scale

How likely are you to doze off or fall asleep during the following situations,
in contrast to just feeling tired?

For each of the situations listed below, give yourself a score of 0 to 3, where
0 = Would never doze; 1 = Slight chance; 2 = Moderate chance; 3 = High chance.

Work out your total score by adding up your individual scores for situations 1 to 8. (If you have not been in the following situations recently, think about how you would have been affected.)

| **Situation** | **Score** |
| --- | --- |
| **Sitting and reading** | 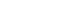 |
| **Watching television** |  |
| **Sitting inactive in a public place (e.g. a theatre/meeting)** |  |
| **As a passenger in a car for an hour with no break** |  |
| **Lying down in the afternoon (when possible)** |  |
| **Sitting and talking to someone** |  |
| **Sitting quietly after lunch without alcohol** |  |
| **In a car, while stopped for a few minutes in traffic** |  |
| **Total score:** |  |

**The Arabic version of the Epworth Sleepiness Scale**

مقياس إيبورث لتقييم الغفــــــوة خلال النهار

**ما احتمالية أن *تغفو أو أن تنعس* في المواقف التالية؟**

**تتراوح الإجابات من إحتمالية عالية للنعاس إلى عدم وجود إحتمالية لذلك. و لكل أجابة درجة تتراوح بين 0 و 3**

**في حالة إذا لم تكن قد مررت بأي من هذه المواقف التالية مؤخراً ,فحاول أن تتخيل كيف كانت ستؤثر فيك.**

|  | **الموقف** | **لا احتمالية أبداً  (0)** | **احتمالية ضئيلة (1)** | **احتمالية متوسطة (2)** | **احتمالية عالية (3)** |
| --- | --- | --- | --- | --- | --- |
|  | الجلوس والقراءة. |  |  |  |  |
|  | مشاهدة التلفاز. |  |  |  |  |
|  | الجلوس ساكناً في مكان عام  (المسرح أو غرفة الاجتماع مثلاً). |  |  |  |  |
|  | كمسافر في سيارة لمدة ساعة بدون توقف. |  |  |  |  |
|  | مستلقياً لترتاح بعد الظهر عندما تسمح الظروف. |  |  |  |  |
|  | الجلوس والتحدث مع شخص ما. |  |  |  |  |
|  | الجلوس بهدوء بعد الغداء. |  |  |  |  |
|  | في السيارة أثناء التوقف لبعض الدقائق أثناء حركة المرور. |  |  |  |  |
